# Innovationsunterstützung im BfArM – Erfahrungen aus den Beratungen zu digitalen Gesundheitsanwendungen (DiGA)

**DOI:** 10.1007/s00103-021-03410-0

**Published:** 2021-09-14

**Authors:** Wiebke Löbker, Anne Christin Böhmer, Barbara Höfgen

**Affiliations:** 1 Leitung Innovationsbüro, Changemanagement, Bundesinstitut für Arzneimittel und Medizinprodukte (BfArM), Kurt-Georg-Kiesinger-Allee 3–5, 53175 Bonn, Deutschland; 2 Bundesinstitut für Arzeimittel und Medizinprodukte, Kurt-Georg-Kiesinger-Allee 3, 53175 Bonn, Deutschland

**Keywords:** Beratung, BfArM, DiGA(‑Fast-Track), Innovationsbüro, Kick-off-Meeting, Advice, BfArM, DiGA (Fast Track), Innovation office, Kick-off meeting

## Abstract

Seit Mai 2020 können Hersteller einen Antrag zur Aufnahme einer digitalen Gesundheitsanwendung (DiGA) in das Verzeichnis nach § 139e Fünftes Buch Sozialgesetzbuch (SGB V) beim für das Bewertungsverfahren zuständigen Bundesinstitut für Arzneimittel und Medizinprodukte (BfArM) stellen. Diesem neuen Antragsverfahren liegen spezifische Anforderungen und Bewertungsparameter zugrunde, zu denen sich herstellerseitig eine Vielzahl an Verfahrens- und wissenschaftlichen Fragestellungen an das BfArM ergibt.

Um diesem Bedarf Rechnung zu tragen, hat das BfArM seine etablierten Informations- und Beratungsangebote gezielt für diese neuen Fragestellungen erweitert. Welche dies bezogen auf DiGA im Einzelnen sind, wo entsprechende Informationen und Unterlagen zu finden sind und was diese jeweils auszeichnet, wird in diesem Beitrag dargelegt. Des Weiteren wird mit Blick auf die zwischen Mai 2020 und April 2021 durchgeführten Beratungsgespräche analysiert, welchen Einfluss diese auf das Ergebnis im jeweiligen Antragsverfahren zur Aufnahme in das DiGA-Verzeichnis haben. Dabei bestätigt sich, dass die frühzeitige Erörterung bewertungsrelevanter Parameter und die Berücksichtigung der Beratungsempfehlungen mit einem positiven Bewertungsergebnis korrelieren: Zu den im Verzeichnis gelisteten DiGA hatten 80 % der Hersteller zuvor eine Beratung durch das BfArM in Anspruch genommen. Die Quote zurückgezogener bzw. abgelehnter Anträge war dagegen deutlich höher, wenn Hersteller zuvor keine Beratung in Anspruch genommen hatten, gegenüber Herstellern, die im Vorfeld zentrale Aspekte mit dem BfArM diskutiert hatten (63 % vs. 35 %). Insgesamt profitieren alle Seiten von dem frühzeitigen, intensiven Austausch – letztendlich insbesondere die Patientinnen und Patienten durch einen unverzögerten Einzug von DiGA in die Regelversorgung aufgrund höherer Antragsqualität.

## Abstract (en)

Since May 2020, manufacturers have been able to submit an application to the Federal Institute for Drugs and Medical Devices (BfArM) for the inclusion of a digital health application (DiGA) in the directory pursuant to section 139e of the German Social Code Book V. This new application procedure is based on specific requirements and assessment parameters for which a large number of procedural and scientific questions arise on the side of the manufacturers.

In order to meet this need, the BfArM has specifically expanded its established information and advisory services for these new questions. This article describes in detail what these are in relation to DiGA, where the corresponding information and documents can be found and what distinguishes them in each case. Furthermore, with a view to the counselling interviews conducted between May 2020 and April 2021, their influence on the outcome of the respective application procedure for inclusion in the DiGA directory is analysed. This confirms that the early discussion of parameters relevant to the evaluation and the consideration of the advisory recommendations correlate with a positive evaluation result. For the DiGA listed in the directory, 80% of the manufacturers had previously received advice from the BfArM. The rate of withdrawn or rejected applications, on the other hand, was significantly higher if manufacturers had not previously sought advice from the BfArM in advance compared to manufacturers who discussed key aspects (63% vs. 35%). Overall, all sides benefit from the early, intensive exchange – in the end especially the patients through introduction of DiGA into standard healthcare due to higher application quality without delay.

## Einleitung

Mit dem Digitale-Versorgung-Gesetz (DVG; [1]) hat der Gesetzgeber als Grundlage für die „App auf Rezept“ ein neues Bewertungsverfahren beim Bundesinstitut für Arzneimittel und Medizinprodukte (BfArM) eingeführt, an dessen Ende nach positiver Bewertung die Aufnahme in das Verzeichnis erstattungsfähiger digitaler Gesundheitsanwendungen (DiGA) steht [2]. Diesem Verfahren liegen, den Eigenschaften der digitalen Produkte Rechnung tragend, neben einem eigenen Antragsverfahren, dem DiGA-Fast-Track nach § 139e Fünftes Buch Sozialgesetzbuch (SGB V), auch neue, spezifische Anforderungen und Bewertungsparameter zugrunde. Zu diesen ergibt sich herstellerseitig naturgemäß eine Vielzahl an Fragen hinsichtlich der zu erfüllenden Voraussetzungen, zum Verfahrensablauf, zu Fristen sowie insbesondere zu vorzulegenden Nachweisen, aber auch viele grundsätzliche Fragen zu DiGA im medizinprodukterechtlichen Kontext.

Hersteller können sich auf freiwilliger Basis im Vorfeld der Antragstellung durch das BfArM beraten lassen (z. T. gebührenpflichtig). Hierfür hat das BfArM seine bereits etablierten, umfangreichen Informations- und Beratungsangebote [3] gezielt für diese neuen Fragestellungen erweitert. Welche dies bezogen auf DiGA im Einzelnen sind, wo entsprechende Informationen und Unterlagen zu finden sind und was diese jeweils auszeichnet, wird in diesem Beitrag dargelegt.

Des Weiteren werden die zwischen Mai 2020 und April 2021 durchgeführten Beratungsgespräche im Zusammenhang mit den nachfolgenden Anträgen zur Aufnahme der DiGA in das Verzeichnis dahin gehend analysiert, welchen Einfluss die Beratungen im Vorfeld auf das Ergebnis im jeweiligen Antragsverfahren haben.

## Beratung durch das BfArM: Frühzeitigen Austausch mit den Herstellern fördern, Antragsqualität erhöhen und sichere und wirksame Innovationen ermöglichen

Auch wenn die Anforderungen an Arzneimittel und Medizinprodukte durch gesetzliche Rahmenbedingungen, ergänzt durch zahlreiche Guidance-Dokumente, klar definiert sind, bleiben häufig viele Fragen offen oder die Vorgaben für spezifische Produkte sind nicht immer eindeutig aus Entwickler- und Antragstellersicht zu interpretieren.

Das BfArM bietet daher Herstellern umfangreiche Unterstützungs- und Beratungsformate für jede Produktentwicklungsphase und zu allen bewertungsrelevanten Aspekten, individuell auf den jeweiligen Beratungsbedarf und Entwicklungszeitpunkt zugeschnitten, an. Diese verfolgen das Ziel, den Austausch zwischen den Expertinnen und Experten der Behörde und den Herstellern zu fördern, damit aussagekräftige Evidenz für Zulassungs- und weitere Bewertungsverfahren ohne unnötige Verzögerungen generiert und Patientinnen und Patienten ein frühzeitiger Zugang zu innovativen, wirksamen und sicheren Arzneimitteln und Medizinprodukten ermöglicht wird.

Frühere Studien konnten zeigen, dass die Inanspruchnahme einer Beratung und insbesondere die Berücksichtigung von Beratungsempfehlungen, z. B. zum Studiendesign, im Arzneimittelbereich mit dem Erfolg von Zulassungsanträgen korreliert [4]. Dabei hat auch der Zeitpunkt der Beratung einen Einfluss: Je früher die Beratung im Entwicklungsprogramm in Anspruch genommen wurde, desto höher war die Erfolgsrate im späteren Zulassungsverfahren [5]. Neben dem Angebot der wissenschaftlichen Beratung auf nationaler Ebene oder zusammen mit der Europäischen Arzneimittelagentur zu konkreten Aspekten, z. B. bzgl. eines Antrags auf klinische Prüfung oder eines Arzneimittelzulassungsantrags, bietet das BfArM daher mit dem Innovationsbüro seit 2017 eine Anlaufstelle für den frühzeitigen Austausch mit Herstellern zu aufkommenden Trends und Produktideen. Es gibt dabei insbesondere Orientierung zu den regulatorischen arznei- und medizinprodukte- sowie sozialrechtlichen Rahmenbedingungen. Zudem werden durch das Innovationsbüro neue Verfahren, wie im aktuellen Beispiel der DiGA-Fast-Track, intensiv durch Informations- und Beratungsleistungen proaktiv begleitet.

## Beratungs- und Informationsspektrum rund um DiGA: Von Orientierungshilfe, Roadshow über Leitfaden zu DiGA-Beratungen

Gerade zu einem neu eingeführten Verfahren wie dem DiGA-Fast-Track ergibt sich auf Herstellerseite eine Vielzahl an Fragen zu Details des Verfahrensablaufs, einzuhaltenden Fristen, zu vorzulegenden Nachweisen mit Blick auf die Anforderungen aus Gesetz und ergänzender Rechtsverordnung (Digitale-Gesundheitsanwendungen-Verordnung, DiGAV [6]), aber auch zu grundsätzlichen Themen rund um DiGA. Deshalb stellt das BfArM auf den jeweiligen Bedarf und Entwicklungszeitpunkt zugeschnittene Informations- und Beratungsangebote zur Verfügung, einschließlich einer die gesetzlichen Vorgaben zusammenfassenden Interpretations- und Lesehilfe in Form eines *Leitfadens* [7]. Darüber hinausgehende, insbesondere produktspezifische Fragen können auf Wunsch des Herstellers im Vorfeld eines Antragsverfahrens im Rahmen eines Beratungsgesprächs bzw. mehrerer Beratungsverfahren mit dem BfArM (*DiGA-Beratung*) erörtert werden.

Erste, eher allgemein orientierende Hinweise gibt das BfArM in den sogenannten Kick-off-Meetings (s. nächster Abschnitt „Beratungsformate des BfArM-Innovationsbüros“).

Bereits vor Inkrafttreten des DVG bzw. der DiGAV ist das BfArM *proaktiv* auf Hersteller, Ärzte und Versicherer zugegangen und hat zusammen mit dem Bundesministerium für Gesundheit (BMG) und dem „health innovation hub“ (hih) des BMG im Rahmen einer *Roadshow* über den Fast-Track und die damit für das Gesundheitssystem verbundenen Möglichkeiten informiert, sodass sich alle Beteiligten bereits im Vorfeld über das Verfahren informieren und entsprechend vorbereiten konnten.

Die 2014 veröffentliche *Orientierungshilfe für Medical Apps* [8], die bei der medizinprodukterechtlichen Einordnung digitaler Anwendungen weiterhilft, sowie ergänzende Informationen, *häufig gestellte Fragen (FAQs)* und *Ausfüllhilfen* zum elektronischen Antragsportal sowie *Checklisten* auf den Webseiten des BfArM [9] runden das Unterstützungsangebot ab.

## Beratungsformate des BfArM-Innovationsbüros

Um dem Innovationspotenzial und dem regulatorischen Kenntnisstand der unterschiedlichen Hersteller gerecht zu werden, bietet das Innovationsbüro ein Spektrum an Unterstützung an. Seit der Gründung des Innovationsbüros hat sich gezeigt, dass Hersteller, je nach Entwicklungsstand ihres Projekts, eine unterschiedliche Begleitung oder Betreuung aus regulatorischer Perspektive benötigen.

### Einfache allgemeine Fragen

Einfache allgemeine Fragen beantwortet das Innovationsbüro daher schnell und unbürokratisch per telefonischer oder einfacher schriftlicher Auskunft. Dieser Service verhilft den häufig noch unerfahrenen Akteuren zu einem ersten besseren Durchblick in regulatorischen Bereichen. Das Innovationsbüro hilft zum Beispiel außerdem, passende Ansprechpartner oder Experten zu finden, auch außerhalb des BfArM.

### Kick-off-Meetings

Mit den sogenannten Kick-off-Meetings bietet das Innovationsbüro ein niederschwelliges Beratungsformat insbesondere für akademische Forschungsgruppen, kleine und mittelständische Unternehmen sowie Start-up-Unternehmen, d. h. eine Zielgruppe, die ihren Fokus eher auf wissenschaftlich-technische Fragestellungen legt und mit regulatorischen (medizinprodukte- bzw. sozialrechtlichen) Themen eher weniger vertraut ist. Das Kick-off-Meeting hat zum Ziel, das Bewusstsein und das Verständnis für die regulatorischen Anforderungen im Arzneimittel- und Medizinproduktebereich zu verbessern. Insbesondere für Start-ups ist es häufig wichtig, sich bereits zu einem sehr frühen Zeitpunkt in ihrer Projektentwicklung regulatorische Unterstützung z. B. zu erforderlichen Verfahrensschritten, zu Studienkonzepten oder zu den einzureichenden Unterlagen für ein Bewertungsverfahren einzuholen.

Die Kick-off-Meetings stellen einen ersten informellen Austausch zwischen den Expertinnen und Experten des BfArM und dem Antragsteller dar. Diese frühzeitige Kommunikation kann helfen, Fragestellungen zu erörtern und Herausforderungen oder Schwierigkeiten zu ermitteln, welche sich im weiteren Verlauf des Entwicklungsprozesses mit Blick auf ein späteres Bewertungsverfahren im BfArM ergeben können. Das Kick-off-Meeting ermöglicht somit also eine effektive Unterstützung vielversprechender Projekte mit innovativem Charakter. Zudem können kritische Aspekte frühzeitig identifiziert und beleuchtet werden und die Vorbereitung einer umfangreicheren, tiefergehenden wissenschaftlichen Beratung erleichtern [10].

### DiGA-Beratung

**Figure 1:**
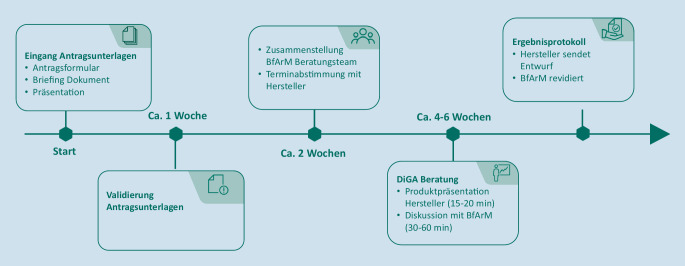

Neben den Kick-off-Meetings bietet das Innovationsbüro [10] seit dem Inkrafttreten der DiGAV DiGA-Beratungen an (Abb. 1). In DiGA-Beratungen werden Fragen im Zusammenhang mit dem DiGA-Fast-Track-Verfahren nach § 139e SGB V mit den Expertinnen und Experten des BfArM diskutiert. Das inhaltliche Spektrum einer Beratung ist weit gefächert und umfasst unter anderem einfache Hilfestellungen bei Verständnisfragen, welche sich nicht durch öffentlich zugängliche Informationen wie dem Leitfaden des BfArM, weiterführende Informationen des Bundesministeriums für Gesundheit oder die FAQs des BfArM beantworten lassen. Auch produktspezifische Fragen können geklärt werden, z. B. zu technischen Details des Antragsverfahrens für eine Aufnahme in das DiGA-Verzeichnis bis hin zu konkreten Fragen zu den vorzulegenden Nachweisen.

Je nach Fragestellung stellt das Innovationsbüro hierfür ein Team aus Expertinnen und Experten unterschiedlicher Fachbereiche des BfArM zusammen, um somit gezielt auf die Fragen der Antragssteller einzugehen. Es wird z. B. gemeinsam erörtert, ob sich aus dem Zweck, der Funktion oder der Umsetzung der digitalen Anwendung gegebenenfalls Fragen in Bezug auf die DiGA-Konformität oder zu der Definition einer DiGA ergeben. Dabei können Hersteller wertvolle Erkenntnisse gewinnen und Hinweise erhalten, die im Rahmen der weiteren Ausgestaltung ihrer Projekte noch einmal überdacht werden können.

Weitere Aspekte einer DiGA-Beratung können die Anforderungen an die Nachweisführung zu den positiven Versorgungseffekten sein. Dies gilt sowohl für Anträge zur vorläufigen als auch zur dauerhaften Aufnahme in das DiGA-Verzeichnis. Im Falle einer angestrebten vorläufigen Aufnahme kann beispielsweise die systematische Datenauswertung oder das Evaluationskonzept Gegenstand der Beratung sein. Bei einer angestrebten endgültigen Aufnahme kann z. B. diskutiert werden, ob die bereits erhobenen Daten für eine endgültige Aufnahme in das Verzeichnis ausreichen oder ob zunächst ein Antrag zur vorläufigen Aufnahme in das DiGA-Verzeichnis infrage kommt.

Um sicherzustellen, dass zwischen dem Hersteller und dem BfArM ein gleiches Verständnis vom Ergebnis der Beratung besteht, erstellt der Hersteller im Anschluss an das Beratungsgespräch ein Ergebnisprotokoll (entsprechend einer Entwurfsvorlage des BfArM) und legt dieses einem späteren Antrag auf Aufnahme in das Verzeichnis bei.

## Beantragung von Beratungen, Kostenabschätzung und Hinweise

Das Innovationsbüro unterstützt und begleitet die Hersteller auch in der Vorbereitung auf Kick-off-Meetings und DiGA-Beratungen. Das übergeordnete Ziel ist dabei, die Beratung für den Hersteller so effizient wie möglich zu gestalten. Das Innovationsbüro bietet unkompliziert eine schriftliche oder mündliche Auskunft mit Blick auf die einzureichenden Beratungsunterlagen und das geeignete Beratungsformat an. Darüber hinaus informiert es auf seiner Internetseite www.bfarm.de/innovation über die Voraussetzungen einer Beratung und bietet Erläuterungen für Antragssteller an [10].

Zusätzlich geht der DiGA-Leitfaden [7] in einem separaten Kapitel auf das Angebot der Beratung und die damit verbundenen Gebühren ein. Die Frage nach den Kosten, welche im Rahmen einer Beratung auf Hersteller zukommen, wird durch die Beschreibung der einzelnen Gebührenkategorien adressiert. Ziel ist hierbei, die Kosteneinschätzung transparent und für den Hersteller möglichst planbar zu gestalten, denn insbesondere für Start-ups ist eine Kosteneinschätzung von hoher Bedeutung. Zur besseren Orientierung und Planbarkeit können Hersteller zudem anhand der eingereichten Fragestellungen eine Einordnung in die entsprechenden Gebührenkategorien beim Innovationsbüro erfragen. Jedoch sollten Hersteller hierbei beachten, dass eine Abrechnung erst entsprechend des dann tatsächlich geleisteten personellen und Sachaufwands erfolgt und sich ggf. Änderungen aufgrund eines im Vorfeld oder im Gespräch herausgestellten zusätzlichen Beratungsumfangs ergeben können.

Grundsätzlich gilt, dass die Beratung jedoch keine Vorabentscheidung eines Antragsverfahrens darstellt. Eine finale Entscheidung zur Aufnahme in das Verzeichnis wird stets nur nach einer intensiven Prüfung der vollständigen Antragsunterlagen getroffen. Der Gegenstand der Beratung ist auch nicht bindend, da dieser auf dem zum Zeitpunkt der Beratung aktuellen Stand der wissenschaftlichen Kenntnis beruht und auf den zur Beratung eingereichten Unterlagen basiert. Weicht ein Hersteller von den Empfehlungen und Einschätzungen des BfArM zu den in der Beratung adressierten Fragen und Themen ab (z. B. in Bezug auf das Studiendesign oder die Durchführung der systematischen Datenauswertung), sind die Gründe antragstellerseitig ausführlich darzulegen.

Kenntnisse und Erfahrungen aus den Beratungen fließen ebenso wie die Erkenntnisse aus den Antragsverfahren zur Aufnahme in das DiGA-Verzeichnis in die Unterstützungsangebote und insbesondere auch in die Weiterentwicklung des Verfahrens ein. Damit wird die Qualität des Verfahrens kontinuierlich und langfristig sichergestellt.

## Erste Erfahrungen aus den DiGA-Beratungen

**Figure 2:**
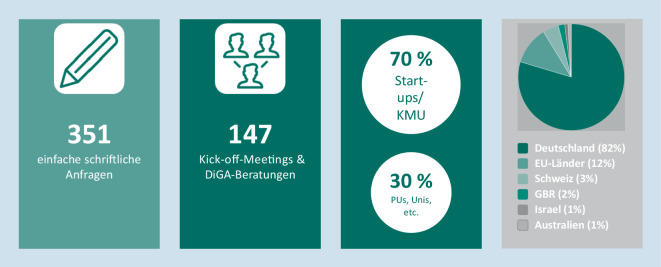

Seit Mai 2020 sind insgesamt 147 Anträge für Kick-off-Meetings und DiGA-Beratungen von 122 Herstellern bzw. Antragstellern sowie 351 einfache schriftliche Anfragen beim Innovationsbüro eingegangen (Abb. 2, Stand 21.04.2021).

**Figure 3:**
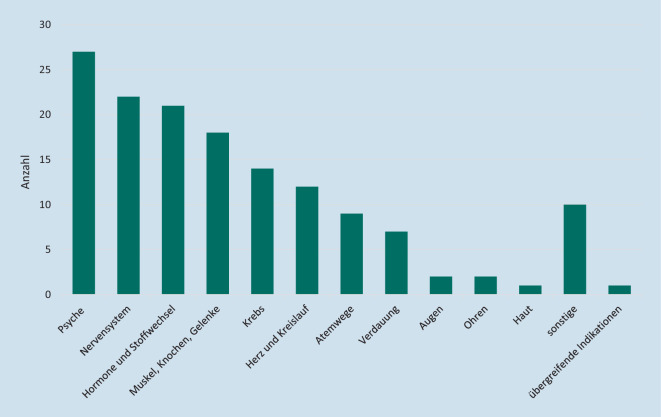

Diese Kick-off-Meetings bzw. Beratungsverfahren bezogen sich am häufigsten auf Produkte aus dem Indikationsbereich „Psyche“, gefolgt von den Bereichen „Hormone und Stoffwechsel“ bzw. „Muskeln, Knochen, Gelenke“ sowie onkologische und das Herz-Kreislauf-System betreffende Produkte (Abb. 3). Somit zeigt sich gegenüber den bisher im DiGA-Verzeichnis gelisteten Anwendungen ein deutlich breiteres Indikations- und Anwendungsspektrum.

Etwa 70 % der Antragssteller sind Start-ups bzw. kleine und mittelständische Unternehmen (Abb. 2). Der überwiegende Teil der Antragssteller kommt aus Deutschland (82 %). Weitere Anträge stammen überwiegend aus anderen Ländern der Europäischen Union (12 %), der Schweiz (3 %), dem Vereinigten Königreich (2 %), Israel (1 %) und aus Australien (1 %).

Die Themen der Beratungen haben sich seit Beginn der Beratungen im Mai 2020 von zunächst eher allgemeinen, wenig detaillierten Fragen hin zu deutlich komplexeren Beratungsinhalten verschoben. So war der Gegenstand der Beratungen zunächst primär auf den Ablauf des Verfahrens, das Antragsprozedere und damit verbundene Fristen sowie einzureichende Unterlagen, Fragen zur Definition einer DiGA und – häufig ohne nähere Daten hierzu vorzulegen – zur Akzeptanz von einzelnen Evidenzparametern, wie z. B. Endpunkten, fokussiert.

Mit zunehmendem Bekanntheitsgrad des Verfahrens und des Leitfadens beinhalten die Beratungsverfahren aktuell häufig sehr konkrete Fragen zu Funktionen und konzeptionellem Design der DiGA, zur Erfüllung der Eigenschaften einer DiGA hinsichtlich der Anforderungen aus § 33a SGB V und zu medizinprodukterechtlichen Aspekten. Weitere spezifische Beratungsthemen betreffen die Anwendung von DiGA in Kombination mit Dienstleistungen, die systematische Datenauswertung bei Planung eines Antrags zur vorläufigen Aufnahme, konkrete Designaspekte vorzulegender Studien inklusive statistischer Planungen und die Eignung von Endpunkten einschließlich geeigneter Fragebögen. Des Weiteren gibt es klinische Fragen zur Übertragbarkeit auf andere Indikationen sowie Fragen zu Datenschutz und Informationssicherheit. Aufgrund dieser spezifischen Fragestellungen werden zunehmend auch weitere Fachkräfte des BfArM in die Beratungen einbezogen, z. B. Expertinnen und Experten für Biostatistik, Datenschutz und Klinik.

Die entsprechenden Erfahrungen aus den Beratungen fließen auch in die Fortschreibung des Leitfadens, z. B. in Form weiterer FAQs oder Präzisierungen der im Leitfaden aufgeführten Beispiele, ein. Erkenntnisse aus den Antragsverfahren führen zu kontinuierlichen Anpassungen auch im Antragsportal, sodass Fragen/konkrete Anforderungen präziser gestellt werden können und ebenfalls Eingang in die Ausfüllhilfe bzw. in die weitere Ausgestaltung des Antragsportals und der Informationen auf den BfArM-Webseiten finden.

Durch den Dialog und die gesammelten Erfahrungen ergeben sich auch Aspekte, die zu Anpassungen technischer oder regulatorischer Anforderungen in den gesetzlichen Regelwerken führen.

### Die Analyse der Antragsverfahren zeigt den Mehrwert einer Beratung

**Figure 4:**
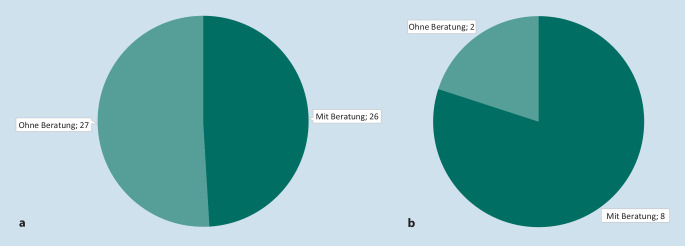

Zum Stand 21.04.2021 haben sich von den 53 Herstellern, die bisher einen oder mehrere Anträge zur Aufnahme einer DiGA in das Verzeichnis nach § 139e SGB V gestellt haben (insgesamt 60 Anträge/Ersteinreichungen, darunter auch laufende Verfahren), 26 Hersteller (49 %) vor der Antragstellung beraten lassen (Abb. 4 und 5). 27 Hersteller (51 %) haben davon keinen Gebrauch gemacht.

**Figure 5:**
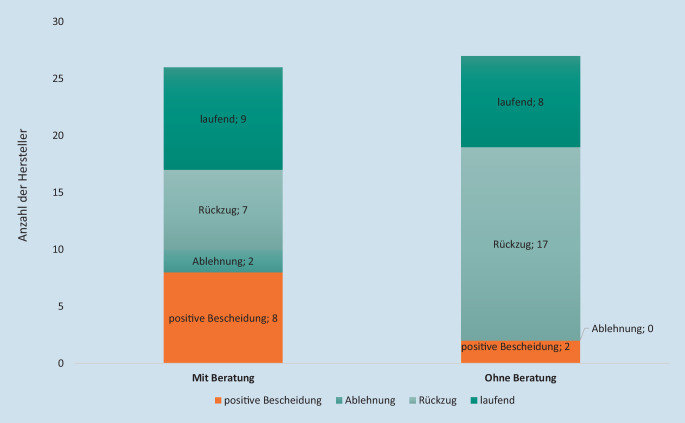

Von den 10 Herstellern, die mittlerweile einen bzw. mehrere positive Bescheide vom BfArM erhalten und damit eine oder mehrere DiGA im Verzeichnis gelistet haben (insg. 12 DiGA), haben 8 Hersteller (von 10 DiGA) im Vorfeld der Antragstellung den Dialog im Rahmen einer DiGA-Beratung mit dem BfArM gesucht, das entspricht 80 %. In diesen Fällen konnte festgestellt werden, dass die Antragsunterlagen größtenteils von hoher Qualität waren und die Empfehlungen aus den Beratungen entsprechend berücksichtigt wurden. Darüber hinaus waren 2 Hersteller mit der Aufnahme ihrer DiGA in das Verzeichnis auch ohne vorherige Beratung durch das BfArM mit ihrem Antrag erfolgreich (20 %).

Von den insgesamt 27 Herstellern, die im Vorfeld der Antragstellung keine Beratung in Anspruch genommen hatten, haben sich im Laufe des Bewertungsverfahrens 17 für eine Rücknahme ihres Antrages bzw. ihrer Anträge zur Aufnahme einer DiGA in das Verzeichnis entschieden, d. h. 63 % (Abb. 5). Dagegen haben nur 7 (27 %) der 26 Hersteller mit vorheriger Beratung ihren Antrag im Laufe des Bewertungsverfahrens zurückgezogen bzw. eine Ablehnung ihres Antrags zur Aufnahme einer DiGA in das Verzeichnis erhalten.

Die Gründe für das Zurückziehen oder die Ablehnung waren mangelnde Evidenz (d. h. unzureichende Aussagekraft der eingereichten Studienergebnisse bzw. bei Anträgen auf vorläufige Aufnahme der systematischen Datenauswertung) oder Mängel bei Datenschutz und Datensicherheit. Zu diesen Aspekten hätte zuvor in einem Beratungsgespräch mit dem BfArM erörtert werden können, ob die Voraussetzungen aus Gesetz und Verordnung hinreichend erfüllt werden oder relevante Daten, technische Voraussetzungen und erforderliche Angaben fehlen.

In den zurückgezogenen oder abgelehnten Fällen, in denen Hersteller zuvor eine Beratung in Anspruch genommen hatten, waren insbesondere die Mängel zu Datenschutz und Datensicherheit jene Aspekte, die im Vorfeld der Antragstellung nicht Inhalt der Beratungen gewesen waren. Auch bei den Ablehnungen aufgrund unzureichender Evidenz war der Mängelgrund entweder nicht Gegenstand der Beratung mit dem BfArM gewesen oder es war den Empfehlungen des BfArM nicht gefolgt worden. So wurde beispielsweise ignoriert, dass die Dauer der Erhebung der Therapiezielerreichung (Endpunkterhebung) zum Nachweis des positiven Versorgungseffektes für eine dauerhafte Aufnahme in das Verzeichnis in der Beratung vom BfArM als zu kurz angesehen worden war.

In 6 Fällen wurden die entsprechenden Anträge bereits wieder neu eingereicht, 4 Hersteller haben sich vor der Neueinreichung für eine Beratung, insbesondere zu den Aspekten, die zur Rücknahme bzw. Ablehnung des Antrags geführt hatten, entschieden.

Insgesamt ist festzustellen, dass die Qualität und Validität der Anträge der Hersteller, die vor Antragstellung eine Beratung in Anspruch genommen haben, deutlich höher ist. Dies spiegelt sich auch in den Anteilen an zurückgezogenen Anträgen von den Herstellern, die eine Beratung in Anspruch genommen hatten bzw. nicht in der Beratung waren, wider (27 % vs. 63 %, Abb. 5).

So können im Vorfeld eines Antragsverfahrens bereits mit dem BfArM Fragestellungen und Herausforderungen diskutiert und ausgeräumt werden, die sich sonst erst im Verfahren klären ließen. Die Erfahrung hat gezeigt, dass die Behebung potenzieller Antragsmängel bzw. Ergänzungen vorhandener Evidenzlücken innerhalb des gesetzlich vorgegebenen Verfahrenszeitraums von 3 Monaten für den Hersteller oft nicht zu bewältigen ist.

Auch das BfArM profitiert von einer frühzeitigen Befassung mit den Produktdetails im Vorfeld einer Antragstellung: So können aufkommende Trends und neue Entwicklungen frühzeitig identifiziert, wissenschaftliche Aspekte und weitere antragsrelevante Details mit dem Hersteller bereits vor Antragstellung offen diskutiert und durch vorherige Kenntnis der Produkt- und Antragsspezifika eine leichtere und schnellere Antragsbearbeitung mit weniger Rückfragen ermöglicht werden.

Eine Beratung ist allerdings kein Garant für einen reibungslosen und erfolgreichen Ausgang des Bewertungsverfahrens, wenn entweder im Beratungsverfahren nicht alle wesentlichen Details des Antragsverfahrens besprochen werden (können) oder Hersteller die Empfehlungen aus der Beratung nicht hinreichend berücksichtigen. Insofern empfiehlt sich für zukünftige Antragsverfahren ein frühzeitiger Austausch mit dem BfArM, um z. B. im Rahmen eines Kick-off-Meetings die Rahmenbedingungen insbesondere für den Nachweis des positiven Versorgungseffektes und entsprechende Grobkonzepte zum klinischen Studiendesign für die weitere Projektplanung zu sondieren und sukzessive zu späteren Zeitpunkten konkrete Details zum Antragsverfahren und zu vorzulegenden Nachweisen näher zu diskutieren. Das BfArM steht hier den Herstellern beratend und begleitend zur Seite, damit innovative Entwicklungen nicht an unnötigen Hürden scheitern, die durch den gemeinsamen Dialog und ein besseres gegenseitiges Verständnis verhindert bzw. minimiert werden können.

Bei der Interpretation der vorliegenden Analyse ist zu berücksichtigen, dass diese aufgrund des erst vor gut einem Jahr implementierten Bewertungsverfahrens mit bisher 60 Anträgen von 53 Herstellern und 12 erfolgreich abgeschlossenen Bewertungsverfahren zunächst als vorläufig anzusehen ist und durch eine weitere Analyse in größerem Maßstab bestätigt werden sollte. Zudem ist eine gewisse Verzerrung dahin gehend nicht auszuschließen, dass Hersteller, die sich besonders gewissenhaft auf den Antrag vorbereiten, auch diejenigen sind, die eher eine Beratung in Anspruch nehmen. Insgesamt zeigt sich aber mit Blick auf den DiGA-Fast-Track ebenfalls ein deutlicher Trend, der Ergebnisse früherer Untersuchungen bestätigt, die den Mehrwert einer Beratung im Vorfeld durch höhere Erfolgsquoten bei Antragsverfahren zeigen konnten [5].

## Fazit

Seit Inkrafttreten des DVG bzw. der DiGAV im April 2020 bietet das BfArM Herstellern im Vorfeld der Antragstellung ihrer „App auf Rezept“ als Voraussetzung für die Erstattung durch die GKV Beratungen an, in denen auf den jeweiligen Bedarf und Entwicklungsstand zugeschnittene Aspekte des Verfahrens mit dem BfArM erörtert und Interpretationsfragen geklärt werden können. Die seitdem konstant hohe Zahl von Anträgen für Kick-off-Meetings und Beratungen sowie von allgemeinen Anfragen an das Innovationsbüro zeigt das große Interesse an dem Verfahren. Das Indikationsspektrum der digitalen Anwendungen, zu denen beraten wird, ist breit. Auch das unterstützende Informationsangebot des BfArM in Form des Leitfadens oder der Roadshow, die auch bereits im Vorfeld des Inkrafttretens des DVG angeboten wurde, wird gut angenommen.

Die Erfahrungen aus den Beratungsgesprächen und Antragsverfahren fließen in die Weiterentwicklung der Informations- und Beratungsangebote wie den DiGA-Leitfaden sowie des Fast-Track-Verfahrens insgesamt ein.

Von dem frühzeitigen und intensiven Austausch können alle Seiten profitieren, wenn verfahrensrelevante Aspekte adressiert und somit auch Herausforderungen frühzeitig gemeinsam identifiziert und herstellerseitig mit Blick auf das Antragsverfahren entsprechend adressiert werden. Dies wurde durch eine Gegenüberstellung der Anzahl der Hersteller, deren Produkt mit (80 %) versus ohne (20 %) vorangegangene Beratung in das DiGA-Verzeichnis aufgenommen wurde, bestätigt.

Insbesondere die Patientinnen und Patienten profitieren durch einen unverzögerten Zugang zu wirksamen und sicheren „digitalen Helfern“. Das BfArM versteht sich durch diese Beratungs- und Unterstützungsangebote als Förderer einer zukunftsorientierten, sicheren und zweckmäßigen Patientenversorgung.

## References

[1] Bundesanzeiger (2019) Digitale Versorgung Gesetz (DVG). Bundesgesetzblatt Jahrgang 2019 Teil I Nr. 49. https://www.bgbl.de/xaver/bgbl/start.xav?startbk=Bundesanzeiger_BGBl&start=%2F%2F%2A%5B%40attr_id=%27bgbl119s2562.pdf%27%5D#__bgbl__%2F%2F*%5B%40attr_id%3D%27bgbl119s2562.pdf%27%5D__1621183551333. Zugegriffen: 21. Apr. 2021

[2] BfArM (2021) Verzeichnis des BfArM für digitale Gesundheitsanwendungen. https://diga.bfarm.de/de. Zugegriffen: 21. Apr. 2021

[3] BfArM (2021) Beratungen durch das BfArM. https://www.bfarm.de/DE/BfArM/OrganisationAufgaben/Beratungsverfahren/_node.html. Zugegriffen: 1. Mai 2021

[4] Regnstrom J, Koenig F, Aronsson B (2010). Factors associated with success of market authorisation applications for pharmaceutical drugs submitted to the European Medicines Agency. Eur J Clin Pharmacol.

[5] Hofer MP, Jakobsson C, Zafiropoulos N (2015). Regulatory watch: impact of scientific advice from the European Medicines Agency. Nat Rev Drug Discov.

[6] Bundesanzeiger (2020) Digitale Gesundheitsanwendungen-Verordnung (DiGAV). Bundesgesetzblatt Jahrgang 2020 Teil I Nr. 18. https://www.bgbl.de/xaver/bgbl/start.xav?startbk=Bundesanzeiger_BGBl&jumpTo=bgbl120s0768.pdf#__bgbl__%2F%2F*%5B%40attr_id%3D%27bgbl120s0768.pdf%27%5D__1621184133912. Zugegriffen: 1. Mai 2021

[7] BfArM (2021) Leitfaden des BfArM zum DiGA-Fast-Track. https://www.bfarm.de/SharedDocs/Downloads/DE/Service/Beratungsverfahren/DiGA-Leitfaden.pdf;jsessionid=966A8025FE641E985F87E5DD6140A4BE.1_cid319?__blob=publicationFile&v=11. Zugegriffen: 1. Mai 2021

[8] BfArM (2021) Orientierungshilfe „Medical Apps“. https://www.bfarm.de/DE/Medizinprodukte/Abgrenzung/MedicalApps/_node.html. Zugegriffen: 1. Mai 2021

[9] BfArM (2021) Webseite des BfArM zum DiGA-Fast-Track. https://www.bfarm.de/DE/Medizinprodukte/DVG/_node.html. Zugegriffen: 1. Mai 2021

[10] Innovationsbüro des BfArM (2021) Erläuterungen für Antragsteller und weitere Informationen. www.bfarm.de/innovation. Zugegriffen: 1. Mai 2021

